# Impact of Self-concept on Preschoolers’ Dental Anxiety and Behavior

**DOI:** 10.15171/joddd.2015.034

**Published:** 2015-09-16

**Authors:** Leila Erfanparast, Ali Vafaei, Azin Sohrabi, Bahram Ranjkesh, Zahra Bahadori, Maryam Pourkazemi, Shabnam Dadashi, Sajjad Shirazi

**Affiliations:** ^1^Dental and Periodontal Research Center, Tabriz University of Medical Sciences, Tabriz, Iran; ^2^Assistant Professor, Department of Pediatric Dentistry, Tabriz University of Medical Sciences, Tabriz, Iran; ^3^Assistant Professor, Department of Pediatric Dentistry, Tabriz University of Medical Sciences, Tabriz, Iran; ^4^Psychologist, Cyber Psychology and Neurofeedback Center, Tabriz, Iran; ^5^Dentist, Private Practice, Tabriz, Iran; ^6^Student Research Committee dentistry, Faculty of Dentistry, Tabriz University of Medical Science, Tabriz, Iran

**Keywords:** Behavior, child, dental anxiety, self-concept

## Abstract

***Background and aims***. Different factors affect children’s behavior during dental treatment, including psychological and behavioral characteristics. The aim of this study was to evaluate the correlation of self-concept on child’s anxiety and behavior during dental treatment in 4 to 6-year-old children.

***Materials and methods.*** A total of 235 preschoolers aged 4 to 6 years were included in this descriptive analytic study. Total self-concept score for each child was assessed according to Primary Self-concept Scale before dental treatment. Child’s anxiety and child’s behavior were assessed, during the restoration of mandibular primary molar, using clinical anxiety rating scale and Frankl Scale, respectively. Spearman’s correlation coefficient was used to evaluate the correlation between the total self-concept score with the results of clinical anxiety rating scale and Frankl Scale.

***Results.*** There was a moderate inverse correlation between the self-concept and clinical anxiety rating scale scores (r = -0.545, P < 0.001), and a moderate correlation between the self-concept and child’s behavior scores (r = 0.491, P < 0.001). A strong inverse relation was also found between the anxiety and behavior scores (r = -0.91, P < 0.001).

***Conclusion.*** Children with higher self-concept had lower anxiety level and better behavioral feedback during dental treatment.

## Introduction

Anxiety and fear in children during dental treatment has been subjected for many studies. Dental anxiety is common among children and adolescence.^1^ The higher levels of anxiety can endanger the overall oral health status with increased rate of different dental problems.^2,3^ On the other hand, dental anxiety could be potentially challenging for the both child and dentist, which can have considerable implication for the child, dental team, and dental service and also hinder child’s cooperation for treatment.^4^ Low cooperative behaviors in children make the dental treatment difficult and may alter the treatment plan. Furthermore, excessive anxiety can cause more pain perception by the child and reduce the child’s motivation to return and attend the necessary dental treatments.^5^ Different factors affect children’s behavior during dental treatment, some of which include temperament, social class, age, and psychological and behavioral characteristics.^6^

Self-concept, also called self-construction, self-identity or self-perspective is a multi-dimensional construct that refers to an individual’s perception of “self” in relation to any number of characteristics, such as gender, sexuality, racial identity, and many others.^7,8^ The self-concept is an internal model which encompasses self-assessments included -but is not limited to- personality, skills and abilities, occupation(s) and hobbies, physical characteristics, and etc.^9^

In the other word, self-concept contains three parts: self-esteem, stability, and self-efficacy. Self-esteem is the "evaluative" component, where one makes judgments about his or her self-worth, which means positive or negative evaluations of the self.^10,11^ Stability refers to the organization and continuity of one’s self-concept. Self-efficacy is best explained as self-confidence and is specifically connected with one’s abilities, unlike self-esteem.^11^

During early childhood self-concept develops and attributes, abilities, attitudes, and the values are established. By age 3 (between 18 and 30 months), children have developed their categorical self, which is a concrete way of viewing themselves in "this or that" label. Young children can also describe their self-concept in simple emotional and attitude descriptions when is asked for it. Early self-concepts are based on easily-defined and -observed variables. Both internal and external variables can affect young children’s self-concept with the emotional development. For example, child’s temperament can affect how they view themselves and their ability to successfully complete tasks. Children with better frustrations and challenges coping capability are more likely to think of themselves as successful, valuable, and good. On the whole, self-concept is the effectiveness in the individual’s behavior, cognition, emotions, academic achievement, happiness, anxiety, social integration, and satisfaction with life.^12,13^

To the best of our knowledge, there is no study to evaluate the impact of self-concept on children’s behavior and anxiety during dental treatment. Hence, the aim of this study was to assess the relation of self-concept with child anxiety and behavior during dental treatment in 4 to 6-year-old children.

## Materials and Methods

This study was carried out in the Department of Pediatric Dentistry, Tabriz University of Medical Sciences. Data were collected between July and November 2012.

### Study Population 

A total of 235 healthy children (119 boys and 116 girls) aged 4 to 6 years old (mean age of 5.4) were included in this study. Children were selected from new patients referred to the department of pediatric dentistry for routine dental treatments. A comprehensive medical and dental history was taken and a treatment plan was established for each patient. The selected children were in complete physical and mental health with no confounding medical history.

### Inclusion Criteria

- First attendance to a dental setting
- No history of post-traumatic stress
- No history of unpleasant experiences in medical settings
- Having at least one decayed mandibular primary molar requiring injection for restorative treatment

### Assessment Scales

1. *Primary Self-concept Scale*

The Primary Self-Concept Scale^14^ is composed of 24 items. Each item depicts at least one child in a positive role and at least one child in a negative role. Before treatment, a simple descriptive story about each illustration was told to the each child. The child was instructed to draw a circle around the person that was most like him/or her. The test was designed to measure the following eight aspects or factors of self-concept: 1) Peer aggressiveness/cooperation, 2) Peer ostracism/acceptance, 3) Intellectual self-image, 4) Helpfulness, 5) Physiological self, 6) Adult acceptance/rejection, 7) Emotional self, 7) Success/no success. The reliability of Primary Self-Concept Scale test was moderate to high -according to test manual- and the concurrent and construct validity of the original test have been evaluated previously. The test was scored to yield a total self-concept score. Content and construct validity of the questionnaire was evaluated by 5 faculty members of pediatric department of dental school (pediatric dental specialists), 2 psychiatrists, and 2 statistics specialists. The reliability was determined through a pilot study involving 20 children attending pediatric ward using Cronbach’s alpha test. Cronbach’s alpha for the questionnaire was calculated 0.87.

1. *Clinical Anxiety Rating Scale*

Clinical anxiety rating scale was used as a behavioral assessment scale of anxiety. Since a 6-point rating scale was used, the scores ranged from 0 to 5 (Table 1).^15^

**Table 1:** Anxiety rating scale

0. Relaxed, smiling, willing and able to converse
1. Uneasy, concerned; during stressful procedure may protest briefly and quietly to indicate discomfort; hands remain down or partially up to signal discomfort; willing and able to interpret experience as requested; a tense facial expression is evident; may have tears in eyes
2. Child appears scared; tone of voice, questions, and answers reflect anxiety; during stressful procedure, may exhibit verbal protest, quiet cry-ing, and tense and raised (but not interfering) hands; child interprets situation with reasonable accuracy and continues to work to cope with anxiety
3. Shows reluctance to enter situation, difficulty in correctly assessing situational threat; pronounced verbal protest, crying; protest out of pro-portion to threat; copes with situation with great reluctance
4. Anxiety interferes with ability to assess situation; general crying is not related to treatment; body movement is more prominent; child can be reached through verbal communication and, eventually with reluctance and great effort, he begins the work of coping with the threat
5. Child out of contact with the reality of the threat; child cries loudly, is unable to listen to verbal communication, makes no effort to cope with threat, and is actively involved in escape behavior; physical restraint is required

1. *Child's Behavior*

The child’s behavior during treatment was assessed according to the Frankl behavior scale, which divides observed behavior into 4 categories: definitely positive, positive, negative, and definitely negative.^16^

### Procedure 

Informed parent’s consent was obtained and the self-concept test of children was conducted by one of the authors. The test administrator explained the children how to complete the questionnaire. All the children were asked to choose a picture that describes him/her after telling a short story about each picture. Then restoration of decayed mandibular primary molar of all the subjects was carried out by one pedodontist. All children were treated at the same specific decorated room for children dental treatment. After application of a topical anesthetic agent for 3 minutes, inferior alveolar nerve block was administered.

A class II cavity was prepared using a high-speed handpiece and an amalgam filling was done. The average duration of treatment session time was 28 ± 5 minutes for each child. Child’s behavior and anxiety during dental treatment were assessed according to the Frankl Scale and clinical anxiety rating scale, respectively. Two different pedodontists blind to the result of self-concept test accomplished the each abovementioned assay.

### Data Analysis

Spearman’s correlation coefficient was used to analyze the correlation between the scores of three scales. P < 0.05 was considered statistically significant. Data were analyzed using SPSS 15.0 (SPSS Inc, Chicago, Ill., USA).

## Results

Spearman’s correlation coefficient analysis revealed that there was a significant moderate inverse correlation between self-concept scores and anxiety (r = -0.545, P < 0.001), which means that an increase in self-concept can result a decrease in anxiety and vice versa.

We also found a significant moderate relation associated with Frankl Scale score and self-concept score (r = 0.491, P < 0.001). Indeed, children with higher self-concept had better cooperation with higher Frankl Scale scores.

A significant strong inverse relation was found between anxiety scores and behavior in Spearman’s correlation coefficient analysis (r = -0.91, P < 0.001), which means increase in anxiety can reduce the child’s cooperation during dental procedures.

### Multivariate Regression Analysis

A regression analysis was conducted to determine the relative importance of self-concept for predicting the anxiety and Frankl scores. Table2 and 3 summarize the results of the regression analysis. Self-concept scores were found to be significant predictors of the child’s behavior according to Frankl Scale (r^2^ = 0.285) and anxiety score on the clinical anxiety rating scale (r^2^ = 0.304) during dental treatment. An increase in self-concept score was associated with decreased anxiety level (β = -0.552) and improved child’s behavior during dental treatment (β = 0.508).

**Table 2:** The relation between self-concept and anxiety based on regression analysis

**Variables**	**B**	**Std. Error**	**Beta**	**R** ^2 ^	**P-value**
**Self-concept**	-0.380	0.038	-0.552	0.304	<0.001
Dependent variable: Anxiety					

**Table 3:** The relation between self-concept and Frankl scores based on regression analysis

**Variables**	**B**	**Std. Error**	**Beta**	**R** ^ 2 ^	**P-value**
**Self-concept**	0.209	0.023	0.508	0.258	<0.001
Dependent variable: Frankl scores					

## Discussion

The present study evaluated the effect of self-concept on children’s behavior and anxiety during dental treatment. The results showed that children with higher self-concept scores may be less affected by stress, exhibiting positive interactions and better cooperation, which is consistent with the results of previous studies.^12,13^ Psychology, education and other social science branches, have shown that self-concept is the bedrock of social and emotional development. Researchers believe that enhancement of self-concept is essential for social and emotional achievements.^17^ There is consensus among researchers on the importance of self-concept on behavior outcomes. Heusman and Eron^18^ showed a correlation between aggressive behavior and low self-concept. In another study, it was shown that improving self-concept was the most effective technique to decrease aggressive behavior.^19^Jerusalem et al^20^ demonstrated that self-concept indirectly influenced the management of stress and it was concluded that self-concept can satisfactorily predict coping skills.

In fact, self-concept as a personality trait influences the behavior. Children with higher self-concept exhibit more cooperative behavior when they interact with others. Social learning theory forms the theoretical foundation for this prediction, indicating that individuals learn how well to perform, how to behave, and how to be viewed by others. Positive expectations result from a sense of competence and a feeling of self-assurance but negative expectations stem from a sense of inadequacy and self-doubt. It is believed that the child whose experience leads to positive expectations develops a more positive self-concept. Due to positive self-concept, a child’s expectations results in lower threats from others’ achievements, leading to more sharing, assistance, and cooperation with others.^19^

Self-concept is directly associated with an individual’s anxiety level as described by Rogers; if a child feels valued and respected, he or she is more likely to grow up with a positive self-image and become self-actualized.^21^

Despite differences in personality traits, like self-concept, all children experience some levels of anxiety in the dental setting, which might be due to the stress-provoking nature of the dental environment. However, in this study, we found a moderate correlation between child’s self-concept and anxiety during dental treatment. Children with different self-concepts did not differ in experiencing or not experiencing anxiety, but the difference was in the level of anxiety, i.e. children with higher self-concept, exhibited a moderate level of anxiety and children with low self-concept demonstrated higher levels of anxiety during dental treatment. Considering this issue statistically, it has caused a decrease in anxiety score variances, revealing a moderate correlation between anxiety and self-concept in children in the dental setting.

Studies have also shown an inverse correlation between anxiety and self-concept, i.e. a high anxiety level is correlated with low self-concept.^22^ A highly-valued and –loved child during childhood will most probably have a positive self-image with fully functioning person and low anxiety levels.^21^

Self-concept and self-esteem have great roles in mental health; as these factors decrease, symptoms of anxiety, depression, loneliness, shyness and being reserved become evident and if such a trend persists, serious problems will ensue. In the dental setting, similar to other anxiety-provoking situations, children with low self-concept may feel less confident and exhibit lower expectations of future success, resulting in higher levels of fear of failure and anxiety.^23^

We observed a significant strong relationship between anxiety and children’s behavior in the dental operatory. Children with more anxiety exhibited more problematic behaviors compared to those with less anxiety. A study showed that 61% of Swedish children with dental fear can react with behavior management problems.^24^ Carrillo-Diaz et al^25^ also concluded that dental visits, as well as dental treatments, can influence cognitive elements associated with dental anxiety in children. The results of the present study are consistent with those showing that disruptive behavior is related with anxiety; studies have shown that children’s anxiety may predict their behavior in the dental office.^26,27^ We speculate that, self-concept may be potentially considered to estimate child anxiety level and subsequent cooperation level during dental treatment. However, generalization is an issue, and for better clinical outcomes, it is suggested that the relationship between subscales of self-concept and child’s anxiety and behavior be evaluated in future studies. Also it has to be mentioned that anxiety has a multifactorial etiology and is affected by factors such as other personal traits, parenting style, mother’s anxiety, etc. However, all these factors cannot be evaluated in one study because of the large number of the variables involved; so the conclusions should be weighed carefully.

## Conclusion

In this study, a significant correlation between children’s behavior and anxiety with total self-concept scores was found. As, lower anxiety level was correlated with higher self-concept scores which may lead to better behavioral feedback during dental treatment. 

## Acknowledgments

This study was supported and funded by Tabriz University of Medical Sciences. The authors thank the staff at the department of pediatric dentistry for their assistance.
